# Interventional Closure of a Ruptured Sinus of Valsalva with a Konar-Multifunctional Device

**DOI:** 10.1055/s-0042-1750411

**Published:** 2022-12-20

**Authors:** Thomas Krasemann, Michiel Dalinghaus, Gert van den Berg, Bas Rebel

**Affiliations:** 1 Department of Pediatric Cardiology, Sophia Children's Hospital, Erasmus MC, Rotterdam, The Netherlands

**Keywords:** sinus of Valsalva aneurysm, rupture, device closure

## Abstract

A 14-year-old girl with trisomy 13 presented with signs of respiratory failure. This was caused by the rupture of a sinus of Valsalva aneurysm into the right atrium. A Konar Multifunctional Occluder was used for closure. This is the first report of this device for this indication.

## Introduction

Sinus of Valsalva aneurysms are caused by congenital deficiencies of either muscle or elastic fibers in the middle layer of the aortic wall, which elongates and progresses to an aneurysm. 1 2 Clinical signs occur upon rupture. Depending on the shunt, patients can present with clinical signs of acute heart failure comparable to acute aortic insufficiency. 1 Reports in childhood are extremely rare. 1

Treatment can be either surgically by direct closure or percutaneously by device closure. 1 3 4

## Case Presentation

A 14-year-old girl with trisomy 13 was admitted with respiratory deterioration. A cardiac murmur was noted. Transthoracic echocardiography revealed a bicuspid aortic valve with an enlarged sinus of Valsalva, with a tunnel-shaped connection toward the right atrium ending close to the tricuspid valve.

A computed tomography scan confirmed the connection of the enlarged and elongated sinus of Valsalva to the right atrium.

The risk of cardiac surgery was considered too high, as it was uncertain if she could be rehabilitated after an operation with sternotomy and postoperative mechanical ventilation.

**Fig. 1:**
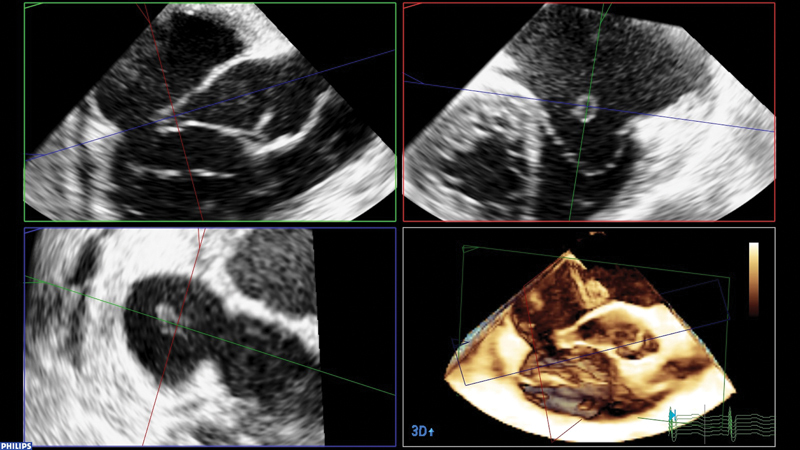
Transesophageal echocardiography delineating the anatomy in different planes and three-dimensional view. Note the elongated sinus of Valsalva and the opening toward the right atrium.

Transesophageal echocardiography under general anesthesia depicted the fistula in detail ( Fig. 1 ).

Three-dimensional echocardiography was used for guidance throughout the procedure close to the fistula, minimizing the use of fluoroscopy.

**Fig. 2:**
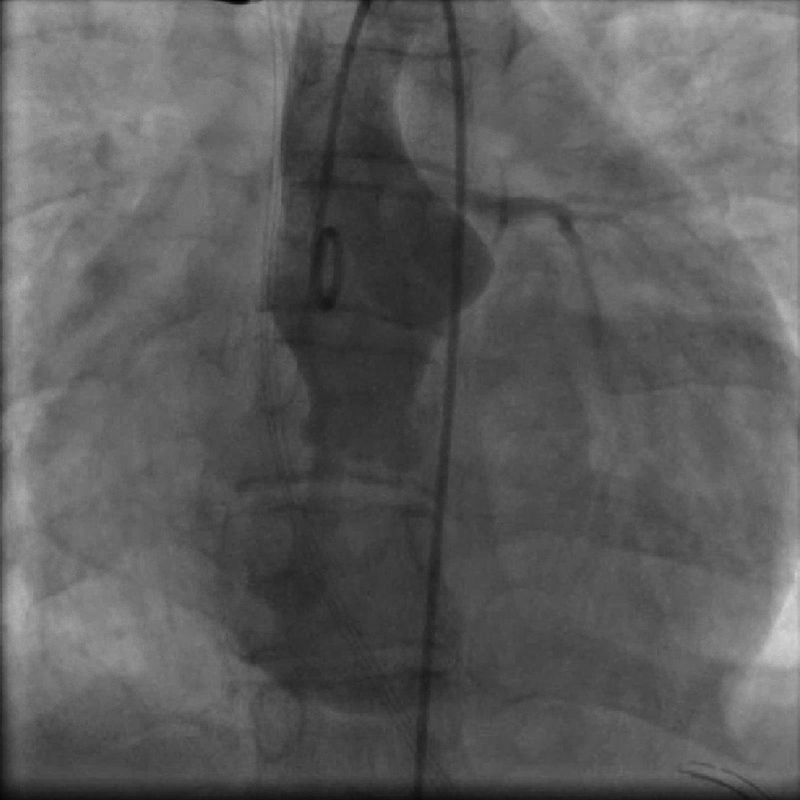
Angiography in the ascending aorta shows the abnormally shaped sinus of Valsalva with a connection to the right atrium.

Aortography showed a very unusual shape of the enlarged sinus of Valsalva ( Fig. 2 ) with a connection to the right atrium of approximately 5 mm.

**Fig. 3:**
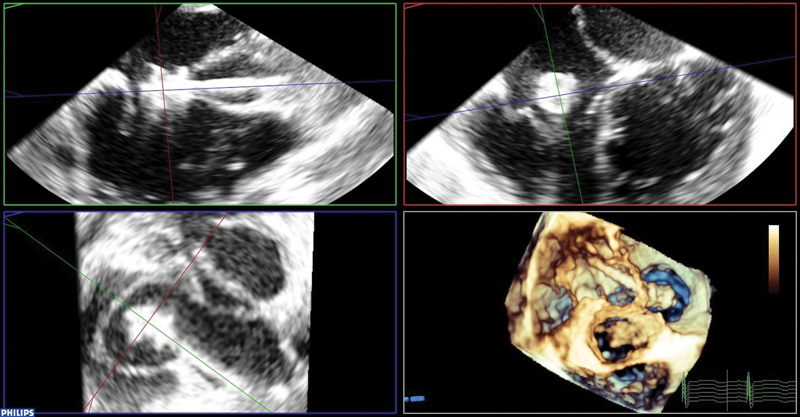
Konar multifunctional device within the ruptured aneurysm, same views as Fig. 1 . The device is still attached to the delivery cable.

**Fig. 4:**
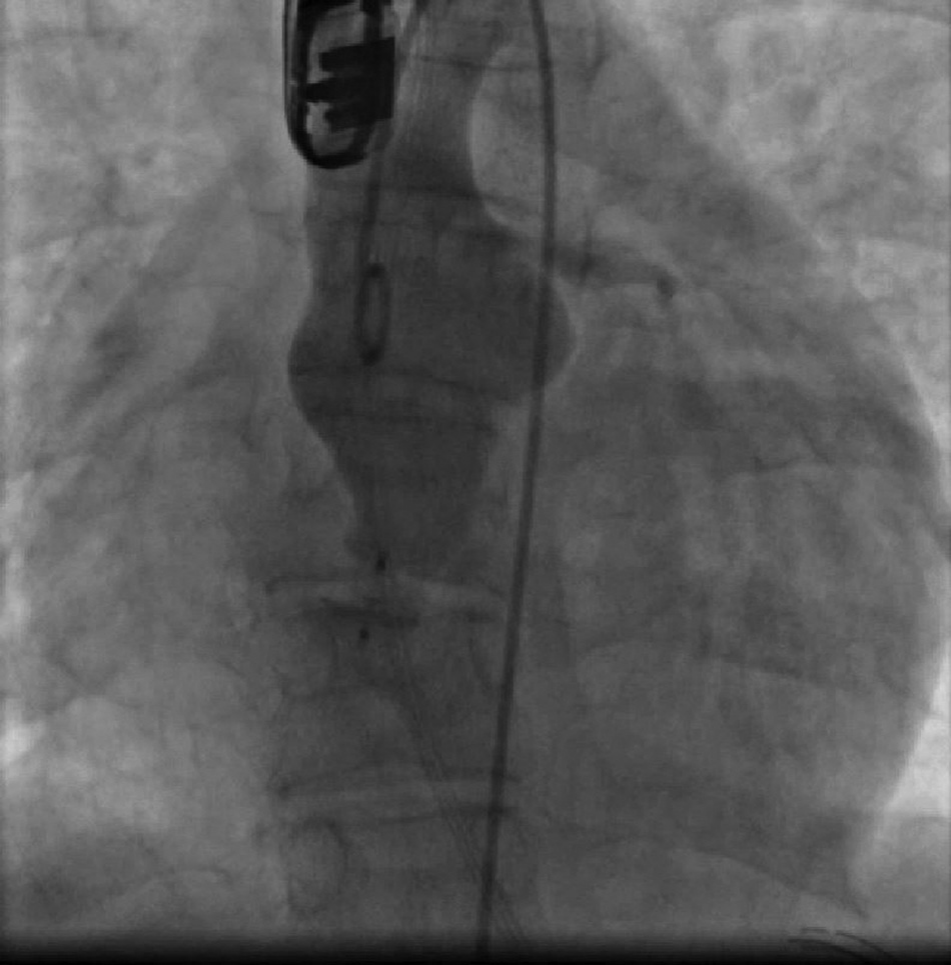
Angiography in the ascending aorta shows the device within the fistula. There is still some flow through the metal meshwork of the device.

This was crossed from the aortic side with a 5F multipurpose catheter (Cordis, Santa Clara, Canada), which was then exchanged with a 5F Launcher (Medtronic, Minneapolis, MN) with an internal diameter of 1.7 mm, which was placed in the inferior vena cava (IVC). Through this, the disk of a Konar Multifunctional Occluder (MFO) device size 8/6 (Lifetech, Shenzen, China) was deployed in the IVC, and then pulled back against the opening of the ruptured sinus of Valsalva. Special attention was paid to the function of the tricuspid valve and the relation between the disk and the leaflets. The body and attached disk of the device were then deployed within the sinus of Valsalva ( Fig. 3 ). A careful wiggle maneuver was performed. Echocardiography and angiography showed adequate positioning. The device was released. Angiography showed the device in a good position with flow through the metal meshwork of the device ( Fig. 4 ).

The girl was extubated 2 hours after the procedure and was discharged 9 days later on aspirin.

Repeat echocardiography 4 weeks later showed minimal flow through the device.

## Discussion

Sinus of Valsalva aneurysms are thought to be rare, even though there are more than 300 reports in the literature. Rupture can lead to acute heart failure, clinically with respiratory deterioration 1 2 as in our patient. The majority of cases reported presented in the third or fourth decade of life. 1 5 with male predominance. 1 In children, rupture of sinus of Valsalva aneurysms is rare. 3 There are reports of syndromic children, one of a boy with mosaic trisomy 13. 6

Most cases arise from either the right- or noncoronary sinus, with a shunt toward the right atrium or ventricle. 1

Treatment consists of closure of the shunt, either surgically or percutaneously with a device. 3 5 7

Different devices for closure include most frequently duct occluders, which are introduced from the venous side 7 with the disk positioned on the aortic side and the device-body in the right heart. Alternatively, ventricular septal defect or other devices can be deployed, depending on the location and size of the fistula. 8 The Konar MF device was able to be deployed from the aortic side, allowing the assessment of tricuspid valve function throughout the delivery process. The body of a duct occluder would have protruded into the right atrium and would have been attached to the delivery cable from this side also. Hence, the device position after release would not have been predictable.

**Fig. 5:**
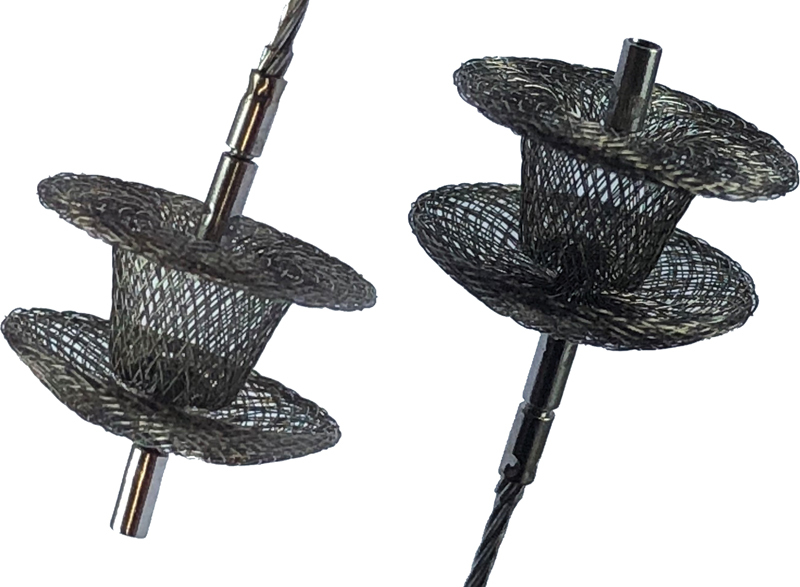
Konar Multifunctional device size 8/6. The device can be attached to the delivery cable from both sides.

The Konar MFO (Lifetech, Shenzen, China) is a double disk device resembling a short (4 mm) duct occluder, with the second disk attached to the body by a small cone-shaped connection. It can be screwed onto the delivery cable from both sides, hence allowing deployment in both directions ( Fig. 5 ). It comes in various sizes. Smaller sizes without any polytetrafluoroethylene polymer membrane can be delivered through a small delivery sheath or catheter, comparable, that is, to Amplatzer vascular plugs, which have also been used to close such defects. 5 In these, the disks and body of the device have the same diameter. Here, a bigger device would have been necessary. Angulation within the aneurysm could have compromised effectiveness.

To our recent knowledge, this is the first report of a Konar MF device used for the closure of a ruptured sinus of Valsalva aneurysm.

Three-dimensional echocardiographic imaging was very helpful in assessing device position, hence minimizing the use of ionizing radiation.

Interventional closure of a ruptured sinus of Valsalva aneurysm with a Konar MF device is feasible. To our knowledge, this is the first description of a Konar MF device used for this indication. Transesophageal guidance is essential to visualize the anatomy in detail and to guide the delivery process.
